# Gold Nanoparticles in Cancer Therapeutics and Diagnostics

**DOI:** 10.7759/cureus.30096

**Published:** 2022-10-09

**Authors:** Shrawani Kulkarni, Sunil Kumar, Sourya Acharya

**Affiliations:** 1 Department of Medicine, Jawaharlal Nehru Medical College, Datta Meghe Institute of Medical Sciences, Wardha, IND

**Keywords:** photodynamic therapy, thermal ablation, diagnostics, drug delivery, cancer, gold nanoparticles

## Abstract

Conventional treatment for cancer is done by chemotherapy, radiotherapy, and/or surgery, which work by inhibiting the growth of the rapidly dividing neoplastic cells and excision of the neoplastic tissue, respectively. These methods affect the healthy tissues along with the diseased ones, as chemotherapeutic agents often cause toxicity in the body and a variety of unfavorable side effects, which can highly impair bodily functions. Otherwise, while operating, there is the danger of some cancerous cells not being excised or unnecessary additional removal of healthy tissues, again causing the patient to lose some functionality of their body. The emerging field of nanotechnology helps to revolutionize imaging and therapeutic management in cancer. The use of metallic nanoparticles, especially those derived from an inert metal like gold, which has a great list of advantages such as high biocompatibility, non-toxicity, easy synthesis, and effective functionality, makes this innovative technology crucial for further propagation of this field. These nanoparticles have a dual function, i.e., helping in precise fluorescent bioimaging and drug delivery of potent medication to specific tissue sites without affecting the general area highly in a negative fashion. Thus, integrating these methods into current clinical practice would advance our methods for diagnostics as well as treatment while decreasing the stress on the patient, overall elevating medical practices.

## Introduction and background

Nanoparticles are part of the many advancements in biomedical research emerging today, which bring in new techniques for imaging, diagnosis, and treatment as well as a variety of other applications. Due to their small size, nanoparticles exhibit enhanced permeability and retention (EPR) effects in tumors, with a relative increase in local tumor concentrations of contrast agents. Among all features of nanoparticles, size plays a particularly important role in tumor imaging. Nanoparticles are effective for drug delivery, i.e., the delivery of medicine to the body, because they can very precisely find diseased cells and carry the medicine to them. This means that one can be treated with a smaller dosage and thereby fewer side effects [1].

An assortment of materials can be used for synthesizing nanoparticles, such as silica and carbon, as well as other polymers and metals like silver, gold, copper, titanium, selenium, and magnesium [2]. Nanoparticles are highly useful because of their decreased size, high reactive action to living cells, heat stability, large surface area to volume ratio, ability to translocate into cells, etc., with metallic nanoparticles being a subgroup that is widely used. Different sizes and shapes of metallic nanoparticles have various optical properties, which can be brought about by different methods of synthesis, which alter the surface chemistry of nanoparticles, helping to visualize images by producing quantum effects that interact with surroundings [3].

Gold has held an evolving focus in the innovation community, even in ancient times when it was mostly used for tinting glass and ceramic ornamentation, which all changed through Faraday’s lecture in 1857 on gold and its properties related to light, bringing it to the forefront for nanoparticle experimentation [4].

In 1925, there was the start of the usage of molecular structures of gold in clinical trials to alleviate rheumatoid arthritis. This was one of the first indications of aurum nanoparticles (AuNPs) in medical therapy [5].

As the research on AuNPs continued, the true leaps and bounds in advancement only occurred after 2005, when gold nanoparticles (GNPs) were established to be one of the most reliable types of nanoparticles. They had the advantages of being easy to prepare, having controllable size and shape, high biocompatibility causing limited toxicity, and most importantly, the property of localized surface plasmon resonance (SPR) [4,6]. The nanoparticles are also extraordinarily adaptable and can undergo a vast amount of surface modification due to advantageous electrical and physical properties present intrinsically, thus, easily undergoing processes such as conjugation. Conjugation is the process of the formation of a chemical bond with various ligands or molecules, which helps in increasing the specificity of the AuNPs to reach target tissues, adding to its large list of advantageous properties [5-7].

The property of SPR in GNPs is so important because visualization occurs in the near-infrared resonant (NIR) imaging region to the visible region on the light wave spectrum, making it extremely easy for us to see these particles, especially with the imaging modalities available to us, making it most cost-effective. Thus, the diagnostic uses of GNPs are a spectrum, with active participation in NIR imaging, magnetic resonance imaging (MRI), photoacoustic imaging, etc. [2,8].

Though GNPs have applications in multiple diseases and pathologies, they have extensive use in cancer "theranostics," ranging from bioimaging and bio-sensing, with negative effects on tumor microenvironments, to drug deliveries and immunotherapy, which are discussed in detail in this review article [7,8].

## Review

Methodology

The following goals are being pursued with this review: GNPs and their use in the diagnosis of cancer and the therapy of cancer.

**Figure 1:**
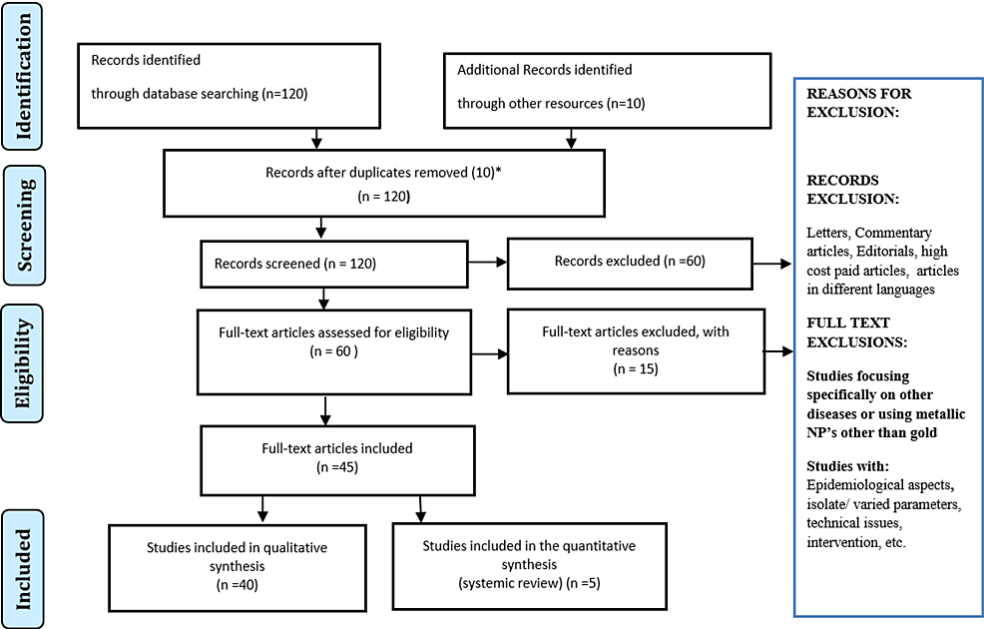
PRISMA methodology PRISMA: Preferred Reporting Items for Systematic Reviews and Meta-Analyses; NPs: nanoparticles.

A literature search in English was conducted using the electronic databases PubMed, MEDLINE, Embase, and Google Scholar. The search terms were as follows: gold nanoparticles [OR] GNPs [OR] AuNPs [OR] cancer diagnosis [OR] cancer therapy [OR] cancer theranostics. The archiving of relevant papers was supported by the writers' personal knowledge and experience in the field. Articles that match the following criteria were included in this review: studies in English, studies from the last 10 years, and studies devoted entirely to GNPs, cancer therapy, and cancer diagnostics. The research methodology by Preferred Reporting Items for Systematic Reviews and Meta-Analyses (PRISMA) method is shown in Figure 1 below.

Synthesis

There is a multitude of ways in which there can be a synthesis of gold nanostructures and where synthesis can occur artificially as well as naturally.

The first gold nanosphere was made by Faraday in 1857 by reducing gold chloride using phosphorus and stabilizing it with carbon disulfide producing a colloidal suspension of gold particles, which today are known as GNPs. Thus, GNPs have always been synthesized by reducing an auric salt followed by stabilization with an agent. The citrate synthesis method is the most common way of production of GNPs and is executed by the reduction of tetrachloroauric acid at different pressures and temperatures using a reliable and common reducing cum stabilizing agent, sodium citrate [9].

The true progress in the field of GNP synthesis started in 2000 when there was a synthesis of one-dimensional, two-dimensional, and three-dimensional as well as hollow Au nanoparticles, which were called anisotropic structures. This particular property of AuNPs facilitates the optical use of the particles allowing the particles to be visualized, but in a safe method with the least amount of absorption in the body [10]. Based on the objective of the research or clinical trial being performed, there can be surface modifications like changes in size and shape performed on the GNPs, which can be achieved by varying temperature, rate of addition of chemical reactants, and concentration of the salt used as a base. Thus, there is a formation of nanoshells, nanocages, nanospheres, and nanorods [9,11].

The synthesis of different types of GNPs can be done relatively easily as compared to others as there is only modification of the surrounding environment to bring about changes in this malleable metal. The most easily produced types are gold nanocages and nanorods. Gold nanocages can be synthesized in many fashions but highly symmetrical GNPs can be produced by the formation of a silica template onto which there can be seeded growth of gold shelling leading to the formation of gold nanocages on the dissolution of the silica template [12]. The templates used can also be made of polystyrene [13]. Meanwhile, gold nanorods, though harder to synthesize but used extensively as they have excellent light surface plasmon resonance (LSPR) because of their longitudinal surface, can also be produced by the seeding method [14]. An example of functionalization of gold nanorods is via salt aging, pH-controlled assembly, ligand conjugation, etc. of DNA with a thiol group attached at one end [14,15]. There can also be synthesis using wet chemistry or classical chemistry to produce single-crystalline rods as well as pentahedrally twinned rods and polycrystalline rods are often synthesized via templated deposition [16].

Environmentally safe methods for the production of GNPs also exist, e.g., by using plant extracts, thus, creating "GREEN" nanoparticles. These methods are also relatively cheaper and produce less contamination, where maintaining the purity of the metals is of utmost importance. A common method used for the synthesis of these environmentally safe nanoparticles is the biogenic reduction of the metal precursors where the chemical reducing agent is replaced with natural products having a variety of properties such as inherent stability and nontoxic capping. Capping is a method of stabilization of the AuNP similar to PEGylation, which is elaborated later on in the review and has the same functions. It can be achieved by causing electrostatic or hydrostatic reactions between GNPs and proteins or by chemical adsorption of thiol groups on the surface of AuNPs [17-19].

Functionalization

The main functionalities of GNPs in cancer theranostics are, namely, bioimaging and drug delivery. There are many optical characteristics of metallic nanoparticles, the most important, however, is the phenomenon of SPR, when there is exposure to an electromagnetic field causing collective oscillation of free electrons present in metals, which can reach a maximum limit at a specific frequency. This means that the oscillating electrons are resonant with the incident wave, that is, both have the same frequency and there is an excitation of plasmonic electrons resulting in the propagation of the initial signal. Hence, metallic nanoparticles can also be called surface plasmon (SP) sensors. However, for cancer diagnostics and therapy, we need the formation of biosensors, specifically affinity biosensors, devices that convert a biological response into an electrical signal. Affinity biosensors are formed by causing a binding reaction between biorecognition molecules (e.g., cell, nucleic acid, and antibody) and analyte (target molecule), which lend to high specificity. The biorecognition molecule is often bonded to a "label" such as a nanoparticle for easy transportation and recognition within the body. We can then quantify the recognition process through transduction using a transducer, which is any device that can turn one form of energy into another. In this particular case, the "transducer" is the metallic nanoparticle as it has the property of SPR, which converts biochemical reactions into measurable electrical signals [20,21].

The SPR band of noble metals is unique as it has a higher density of electrons, which are free to move and can be attuned easily to the near-infrared region as absorption of waves from the NIR region and visible light region is higher in gold. Therefore, it can be used for near-infrared resonance imaging modalities such as MRI, photoacoustic imaging, and electroencephalography (EEG), which are commonly available [22,23].

Bioimaging occurs through the processes of absorption and scattering, mostly Rayleigh and Raman scattering, which are elicited by using GNP giving higher-resolution images for cancer detection. This occurs through the aforementioned LSPR phenomenon, specifically for spherical nanoparticles and nanorods, which are easier to convert into biosensors, and the biological reaction between the analyte and the biorecognition element causes reactions that change the refractive index close to the surface of the nanoparticle activating SPR property producing electric signals which can be used to visualize the particles. This same concept also gives rise to the newer technique of photothermal therapy (PTT) [24].

Conjugation of nanoparticles with ligands helps with stabilization and performs several surface modifications influencing tissue specification, uptake by tissues, and cytotoxicity. The differing ratios of the addition of ligands and nanoparticles help in targeting different tissues and in such a way can also be used for drug delivery and other transport. The process of conjugation can occur through covalent or noncovalent bonding. The covalent methods cause a modification of the surface via replacement, which modifies surface chemistry. The noncovalent methods are dependent on weak interactions between molecules, which do not modify the surface chemistry [25].

Fluorescence imaging is the basis of conducting cancer imaging as it inculcates near-infrared emissions, which have a wavelength of 700 to 900 nm, for noninvasive techniques of imaging. There is effective visualization of structures present both in vivo and in vitro, as these gold molecules can easily absorb light at wavelengths below 1000 nm. The photons from such NIR emissions can penetrate deep into the tissue matter giving information about the structure and functionality without causing any degeneration to the internal matter of the body. The usage of nanoparticles, especially those that are biologically produced, overcomes several hurdles like a large scattering of photons, cell toxicity, decreased sensitivity, and issues with tissue penetration generated by using fluorophores [26]. For example, if we focus on nanoclusters that are electron-dense, fluorescent, and ultra-small in nature, they can penetrate effectively to get special and temporal images. These nanoclusters are synthesized using gelatin as a stabilizing agent with alterations mediated through glutathione, there is the addition of silver nanoparticles, to increase the fluorescence. This combination can be easily visualized, especially in the skin where due to linear unmixing there can be synchronous viewing of skin and the AuNPs [27]. Another example is the usage of histidine as both the reducing and capping agent in nanoclusters resulting in a bluish-green fluorescence, which was enhanced by conjugation to a NIR organic dye for in vivo imaging visualized through multiphoton absorption (MPA) [28].

Thus, a large number of different types of AuNPs can be synthesized for fluorescent imaging using a variety of stabilizing agents and adapting primary capping agents with other functional groups like disulfides or alkane thiols [29].

Hence, by remolding the AuNPs, traditional methods like MRI and CT can be augmented with elevated applications not just restricted to imaging but also lending to therapy. Studies have been done where GNPs had substantial MRI and magnetic hyperthermia therapy outcomes in both subcutaneous and deep tissue tumors, indicating broad-spectrum utilization of GNPs in cancer diagnosis. This particular type of nanotechnology is also being used in positron emission tomography (PET) imaging as well [30].

The photoacoustic technique is another emerging noninvasive imaging modality where there is an attachment of antibodies to nanoparticles of smaller sizes around 5 nm. These were able to penetrate tissues and provide the ability to visualize cancer cells while also reducing cytotoxicity as they are removable by excretion through kidneys due to their microscopic size [31].

Gold is very biocompatible; however, this property is highly dependable on size, structure, and the chemical components present alongside the GNPs. As the particles enter the body, there is often the formation of a complex known as the protein corona complex, which is highly complicated in its structure. The particles in this complex have the presence of opsonin on the surface, which is recognized by immune cells present in the body. This causes an onset of immunological reactions in the body, which reduce the biocompatibility of nanoparticles in the body, leading researchers to find ways to subtly introduce the particles into the body without triggering a cascade of immune reactions, which in turn causes reduced internalization of the GNP. To combat this issue, a process called PEGylation is done where a layer of polyethylene glycol (PEG) is put over nanoparticles, which allows these nanoparticles to enter cells without immune cell recognition, thus allowing better internalization. However, complete avoidance is rarely achieved. This technique of imaging is valuable as it helps detect early-stage tumors and leads to precise therapy without allowing the loss of healthy tissue. Currently, surgeons, due to limitations in imaging and the current nonavailability of technology, must perform surgeries without complete precision, often resulting in either leaving cancerous cells behind, causing recurrence, or removal of vital tissues causing loss of functionality. These nanoparticles provide very specific imaging resulting in excellent visualization helping the surgeon perform the procedure with complete reliability, ultimately bringing about a better prognosis for the patient [32].

Therefore, this amalgamation of different types of synthesis and along with a variety of stabilizing processes, capping processes, and PEGlycation help in the formation of AuNP probes, which are highly biocompatible with excellent photo-physical characteristics and those that are tuned to emissions from the visible to near-infrared ranges at both in vitro and in vivo stages [33].

Cancer therapy

Cancer is widespread as well as a major cause of morbidity and mortality. It causes around 8-10 million deaths each year worldwide. Among these deaths, approximately 19.3 million new cases are reported each year. Hence, there is a current and immediate need for the effective treatment of cancer. The treatments available today are still largely conventional methods consisting of surgery involving large numbers of radical procedures where a patient can often lose a function in particular aspects, chemotherapy, and radiotherapy, which are harsh on the patient and often destroys large amounts of healthy tissue as well as other procedures such as immunotherapy and hormone therapy [34].

Conventional chemotherapy is highly effective; however, it also has severe side effects. There is nonselective uptake of damaging chemotherapeutic drugs into both healthy and dysplastic cells present in tissues and organs causing toxicity. Drastic improvement has been seen in the immediate past years with the advent of nanomedicine. It provided an important addition to chemotherapy as a new intervention by acting as a drug delivery agent not causing widespread destruction but due to high specificity helping in the preservation of normal tissue [35].

Doxorubicin (DOX) is a predominant and persistently used antineoplastic drug; however, it is likely to precipitate drug resistance in tumor cells. In some studies, DOX is conjugated with AuNPs that are altered using stabilizers, through either noncovalent or covalent interactions. It also has the chance of avoiding drug resistance in the case of conjugation. Studies also suggest that there is the presence of a connection favoring the intracellular accumulation of the DOX in drug-resistant cancer cells. For example, initially, the AuNPs were PEGlycated along with NH2 with DOX grafted onto this combo, which was done to overcome multidrug resistance, which was effective [36]. In recent times, there is an advancement where there is a synthesis of magneto-gold-fluorescent nanoparticles (MGFs-LyP-1), using a solvent-mediated procedure and these particles elicited genuine autophagy by inducing the formation of autophagosomes. They were used synergistically with DOX. They amplified chemotherapy at nontoxic concentrations by boosting autophagy flux resulting in minimal impairment of vital organs of the mice it was tested on [37]. Hence, drug delivery continues to become more sophisticated with advancements concentrating on eliminating resistance.

The key point for the popularity of GNPs in drug delivery is their easy synthesis due to surface modifications with an array of ligands helping in targeted and specific delivery. Physicochemical properties are characteristic of photodynamic therapy, contrast imaging, as well as thermal ablation, as discussed further in the review. Thus, engineered nanoparticles can detect disease early. All these qualities require us to have a multifaceted approach to GNPs due to their intrinsic multifunctionality [38].

Advancement of cancer management to include nanotechnology will help in early diagnoses, pinpoint therapy such as PTT with reduced limitations, and reduce outrageous treatment costs, making it affordable to the public en masse [39].

PTT is used as a standalone therapy as it causes specific hyperthermia and with the range of different types of GNPs synthesized, we can incite tumor ablation. An added benefit is that there is an accumulation of the nanoparticles within the cells, causing retained action in the surrounding tumor environment [37]. The bigger AuNPs have better optical properties while the smaller injected particles have consistent biological effects as they have a firmer attachment to diseased cells [40]. However, uses of PTT are limited as it does not affect metastatic lesions or an extensive surface area. Thus, there is imbibition of other procedures such as ion radiotherapy and other secondary therapeutic methods [41]. Ion radiotherapy refers to the use of ion beams as the radiation source. These beams contain ions of either hydrogen and helium, which are “light ions,” or carbon and/or oxygen, which are referred to as “heavy ions.” These ions help in killing off the cancerous cells but often damage the normal tissue too. However, with the use of GNPs as radiosensitizers, they enhance the effect of the radiation in only specific tissues and can cause increased deposition of radiation in cancerous areas [42]. AuNPs have large interactivity with X-ray radiation up to 1 MeV as well as with ion radiation. Monte Carlo simulations showed probable radiation sensitization with AuNPs. It was found that the radiation beam will give a lower dose after having passed through the AuNP-containing region. Thus, it increases the therapeutic ratio [43].

Nanoparticles perform a dual function while being internalized into the body, helping target specific tissues for imaging and simultaneously performing several therapeutic functions. The tumor microenvironment (TME) is made up of an accumulation of various cells and an extracellular matrix (ECM) with abnormal vasculature. The ECM has distorted function and appearance resulting in a hypoxic state of the TME. There is also the presence of fibroblasts, which make up a large portion of the TME and have notable effects on tumor progression and therapy. As a result of aberrant vasculature, the microenvironment is in a state of perpetual hypoxia leading to the onset of an immunosuppressive state by the accumulation of immunosuppressive cells secreting immunosuppressive factors, which end up inhibiting dendritic cells, the most important antigen-presenting cells in the body. This produces macrophages with abnormal phenotype, which in turn results in abnormal fibrosis [44]. GNPs are immunostimulatory causing several different types of reactions while interacting with multiple different types of cells. They also have the ability to bring out certain antibody responses to particular antigens when conjugated with the correct type of molecule, thus it is often used as a vaccine adjuvant. This property can be an advantageous factor in cancer immunotherapy [45].

Another alternative is photodynamic therapy, which was first proposed more than 100 years ago. Nowadays, it is a relatively safe and efficient therapeutic intervention working through the production of reactive oxygen species (ROS), especially for skin pathologies [46]. The overproduction of ROS triggers stress that is oxidative in nature. It causes cell death by cell apoptosis or necrosis. At an in-depth look, GNPs also have an apoptotic effect on the tumor itself, causing DNA fragmentation and cell shrinkage, etc., finally culminating in the formation of apoptotic cells, which are phagocytosed by macrophagic cells later [47].

The oxidative stress is brought about by the attachment of GNPs to photosensitizers, which overcomes the multiple drawbacks of using a lone photosensitizer. The drawbacks of photodynamic therapy without GNPs are an aggregation of photosensitizers in bodily media affecting the yield of singlet oxygen as well as the nonspecific distribution throughout the body. The stability of these photosensitizers is ensured by attachment to an AuNP, which also gives them high cell specificity, helping photodynamic therapy given become highly effective (Table 1) [47].

**Table 1:** Examples of different AuNPs used in the therapy of distinct cancers Adapted from [45]. AuNPs: aurum nanoparticles; DOX: doxorubicin; DOX-PECAuNP: doxorubicin-loaded pectin gold nanoparticles; PEG: polyethylene glycol; FA: folic acid; FU: fluorouracil; DTX: docetaxel; HA: hyaluronic acid; K: kaempferol; LIN: linalool.

Anticancer drug	Nano complex name	Main outcome	Anticancer application
Doxorubicin	DOX-PECAuNPs	Stronger cytotoxicity compared to free DOX	Targeted delivery of DOX to hepatocarcinoma cells
5-fluorouracil	AuNPs-PEG5-FU-FA	Increased cytotoxic effects as compared to free 5-FU and FA	
Docetaxel	DTX@HA-clAuNPs	Increased cytotoxicity and tumor inhibition efficacy than free DTX under near-infrared laser irradiation	Targeted anticancer therapy in combination with laser treatment
Kaempferol	K-AuNPs	Increased cell apoptosis, antiproliferative ability, and inhibition of angiogenesis compared to pure kaempferol	Human breast cancer therapy
Linalool	LIN-AuNPsCALNN	Increased antioxidant activity and anticancer activity as compared to linalool and AuNPs alone	Human breast cancer therapy

## Conclusions

AuNPs have become popular due to their increasing pros. These include great biological compatibility, uncomplicated synthesis for different sizes, and easy surface modification causing increased functionality due to the attachment of ligands required to target cancer cells. However, the most important part of gold nanoparticles is the versatility of their applications right from the synthesis of the materials to the applications in therapy. Though we have focused on cancer therapy, these nanoparticles can be used for a multitude of diseases, elucidating their multifunctionality. With nanotechnology, highly individualized medicine for each patient can be delivered, and with even further enhancements, we can take diagnostics and therapeutic procedures to greater efficiency. The processes which are undergoing clinical trials often show a large amount of success, therefore, advancements should be made available to the masses with increased regularity as it could change our medical practices.
